# Interferon Lambda Signaling Restrains Experimental Autoimmune Encephalomyelitis

**DOI:** 10.3390/biomedicines12030526

**Published:** 2024-02-26

**Authors:** Mohammad Asif Sherwani, Samuel J. Duesman, Zdenek Hel, Chander Raman, Nabiha Yusuf

**Affiliations:** 1 Department of Dermatology, University of Alabama at Birmingham, Birmingham, AL 35294, USA; sherwani@uab.edu (M.A.S.); sduesman@uab.edu (S.J.D.); 2 Department of Pathology, University of Alabama at Birmingham, Birmingham, AL 35294, USA; zdenekhel@uabmc.edu

**Keywords:** interferon lambda, multiple sclerosis, neutrophils

## Abstract

IFN-λ is a type III interferon (IFN) with pleiotropic functions in modulating immune responses. To address its function in autoimmune neuroinflammation, we evaluated the development and progression of experimental autoimmune encephalitis (EAE) in IFNLR1 KO *(Ifnlr1^−/−^)* and C57Bl/6 (WT) mice following immunization with MOG_35–55_ peptide. The results show that *Ifnlr1^−/−^* mice developed significantly more severe EAE than WT littermates with a similar day of onset, suggesting the potential of IFN-λ in reducing disease severity. We next interrogated whether IFN-λ differentially modulates EAE induced by encephalitogenic Th1 cells or Th17 cells. Encephalitogenic Th1 or Th17 generated from WT donors were transferred into WT or *Ifnlr1^−/−^* recipient mice. Whereas encephalitogenic Th1 cells induced more severe EAE in *Ifnlr1^−/−^* than WT recipients, the disease severity induced by encephalitogenic Th17 cells was similar. Additionally, in vitro experiments showed that *Ifnlr1^−/−^* macrophages promoted the expansion of myelin peptide-reactive Th17 cells but not Th1 cells. Early in the disease, the spinal cords of EAE mice displayed a significantly greater proportion of Ly6C^−^Ly6G^+^ cells with CXCR2^+^CD62L^lo^ phenotype, indicating activated neutrophils. These findings suggest that IFN-λ signaling restrains activation and migration of neutrophils to the CNS, potentially attenuating neutrophil-mediated disease progression in autoimmune neuroinflammation. Recombinant IFN-λ can be used as a potential therapeutic target for treatment of patients with multiple sclerosis as it has fewer side effects due to the restricted expression of its receptor.

## 1. Introduction

Type III interferons (IFNs), including IFN-λ1 through λ4, signal via a heterodimeric receptor complex consisting of interferon lambda receptor 1 (IFNLR1 or IL-28Rα) and interleukin-10 receptor 2 (IL-10R2) [1]. IFNLR1 is expressed on epithelial cells, neutrophils, dendritic cells, macrophages, and B cells [2,3]. IFNLR1 selectively binds type III IFNs, whereas IL10R2 is shared with other members of the IL-10 family. From a canonical signaling standpoint, signaling through the type I IFNR (IFNAR) and type III IFNR (IFNLR) are very similar in that both activate the Janus kinases (JAKs), tyrosine kinase 2 (TYK2) and JAK1, and the signal transducers and activators of transcription (STAT1 and STAT2) [3]. However, there are distinct differences in the outcome, dependent on cell type and tissue and the level of expression of IFNLR [3,4,5,6]. IFN-λ signaling in neutrophils is predominantly regulatory through mechanisms that include the inhibition of reactive oxygen species (ROS), NETosis, IL-1*β* expression, cell migration, expression of the transcription factor IRF1, and others [3,4,5,6,7,8,9,10]. The regulatory role of IFN-λ signaling in neutrophils has been reported in several autoimmune disorders, including multiple sclerosis (MS), systemic lupus erythematosus (SLE), and rheumatoid arthritis (RA) [3,7,11,12,13]. Recent research demonstrated the role of IFN-λ in the pathogenesis of central nervous system (CNS) autoimmune disease [14]. In the experimental autoimmune encephalomyelitis (EAE) model of MS, *Ifnlr1^−/−^* animals exhibited less severe clinical disease and decreased spinal cord axonal injury compared with wild-type (WT) animals. The underlying biological mechanism was attributed to myeloid cells [14].

To investigate the role of IFN-λ in EAE, we induced EAE in *Ifnlr1^−/−^* mice. Loss of IFNLR resulted in severe EAE in mice in comparison to their WT littermates. The effect was dependent on IFNLR1 expression on non-T cells. We found that specific neutrophil populations contributed to the development of autoimmune inflammation in the EAE model. Overall, the presented data indicate a role for IFN-λ in restraining neutrophils in the EAE model.

## 2. Materials and Methods

*Mice*. C57BL/6 mice were purchased from Jackson Laboratory (Bar Harbor, ME, USA) and bred at UAB. IFNLR1 knockout (KO) mice (*Ifnlr1^−/−^*) were provided by Dr. Herbert Virgin (Washington University School of Medicine, St. Louis, MO, USA). All animals were housed and treated in accordance with the National Institutes of Health guidelines. The approval number from the UAB Institutional Animal Use and Care Committee (IACUC) is 20631.

*EAE induction*. Age- and sex-matched wild-type (WT) C57BL/6 and *Ifnlr1^−/−^* mice were induced for EAE as described previously [15]. The emulsion, composed of 150 μg of MOG35–55 peptide (sourced from GL Biochem, Shanghai Ltd., Shanghai, China) and complete Freund’s adjuvant (CFA) containing 500 μg of *Mycobacterium tuberculosis* (BD), was prepared using a homogenizer (Thermofisher Scientific, Waltham, MA, USA) operating at a maximum speed of 30,000 RPM for 1 min and 30 s.

Briefly, mice were immunized with 150 μg of MOG_35–55_ peptide (MOGp) (GL Biochem, Shanghai Ltd.) emulsified in complete Freund’s adjuvant (CFA), followed by an intraperitoneal injection of 200 ng of *Bordetella pertussis* toxin (Difco Laboratories, Franklin Lakes, NJ, USA) in phosphate-buffered saline (PBS) on the day of immunization and on day 2. Mice were scored daily to assess clinical symptoms of EAE for 30 days. The EAE scoring system ranges from a score of 0 to 6 as described previously [15,16].

### 2.1. Passive Transfer of Encephalitogenic Th1 and Th17 Cells

For passive induction of EAE, MOG_35–55_-reactive Th1 or Th17 cells were generated from C57Bl/6 mice as recently described [15]. Briefly, donor C57BL/6 WT mice were immunized with 150 µg MOG_35–55_ in CFA containing 500 μg of *Mycobacterium tuberculosis* (BD). No pertussis toxin was administered. Ten days following immunization, cells from peripheral lymph nodes and spleen were restimulated with 10 μg/mL MOG_35–55_ under either Th1 or Th17 polarizing conditions for 3 days. Th1 polarizing conditions consisted of 10 ng/mL IL-12 (BioLegend, San Diego, CA, USA) and 0.5 μg/mL anti-IL-4 (BioLegend) in complete Iscove’s modified Dulbecco’s medium (IMDM). Th17 polarizing conditions consisted of 20 ng/mL IL-6 (Tonbo Biosciences, San Diego, CA, USA), 20 ng/mL IL-23 (Bio-Legend), 1 ng/mL TGF-β1 (Tonbo Biosciences), 10 μg/mL anti-IFN-γ (BioLegend), and 0.5 μg/mL anti-IL-4 (BioLegend) in IMDM complete culture media. The differentiated cells were harvested and pooled and dead cells were removed using a Ficoll gradient. CD4 T cells were enriched using magnetic dynabeads (Thermo Fisher, Waltham, MA, USA). These cells were used as “donor cells”. WT and *Ifnlr1^−/−^* mice were injected with 2.5 × 10^6^ cells (Th1 or Th17) resuspended in 500 μL of PBS via intravenous tail injections. Recipient mice were also intraperitoneally administered 200 ng of the pertussis toxin, the same as for active EAE, and they were scored daily to assess clinical symptoms of EAE for 30 days as described above [15].

### 2.2. Isolation of Peritoneal Macrophages and Co-Culture with CD4+T Cells

Thioglycolate (3%) injection (1.5 mL) was given intraperitoneally and, after 72 h, the mice were sacrificed and injected with chilled PBS in the peritoneum. The peritoneal exudate was collected and seeded onto non-tissue culture 100mm dishes in RPMI media with 10% fetal bovine serum (FBS) for 30–45 min to allow macrophages to adhere to the dishes. Subsequently, non-adherent cells were removed by washing twice with Hank’s balanced salt solution (HBSS). Adherent cells were lifted from culture dishes using cold (4 °C) HBSS with 10mM EDTA for 3–4 min. Purified CD4+ T cells from WT and *Ifnlr1^−/−^* MOGp-immunized mice were cultured at 1:3 ratio (macrophage/CD4 T cells ratio) with 20 ng/mL MOGp under Th1 or Th17 polarizing conditions for 72 h and analyzed for IL-17 and IFN-γ expressing cells by flow cytometry [15].

### 2.3. Flow Cytometric Analysis of Th1- and Th17-Polarized MOG-Specific T Cells

IFN-γ and IL-17-expressing cells were enumerated by flow cytometry as described previously [15,16]. Briefly, the cells were stimulated with PMA (50 ng/mL, Sigma Aldrich, St. Louis, MO, USA) and ionomycin (500 ng/mL, Sigma Aldrich) in the presence of brefeldin A (BioLegend) for 4 h at 37 °C. The cells were stained with Zombie NIR live/dead stain, followed by anti-CD4, anti-IFN-γ APC BV650, and anti-IL-17A AF488 (BioLegend, San Diego, CA, USA) antibodies. Flow cytometry data were acquired using an Attune NxT flow cytometer (Thermo Fisher) and analyzed using FlowJo-v10.9.0 software (BD).

### 2.4. Flow Cytometric Analysis of Neutrophil Populations

Mice immunized for active EAE, as described above, were deeply anesthetized on day 10 [15,17]. The mice were perfused and the spines were removed. The spinal cords were dissected and the cells were dispersed without enzymatic digestion and stained with antibodies to Ly6C, Ly6G, CXCR2, CD11b, and CD62L (BioLegend).

### 2.5. Statistical Analysis

The data are presented as means ± SEM. Statistical significance was determined using non-parametric (EAE) or parametric (in vitro experiments and cell populations) two-tailed Mann–Whitney *t*-test.

## 3. Results

### 3.1. IFN-λ Signaling Is Necessary to Restrain the Development of Severe EAE

**Figure 1:**
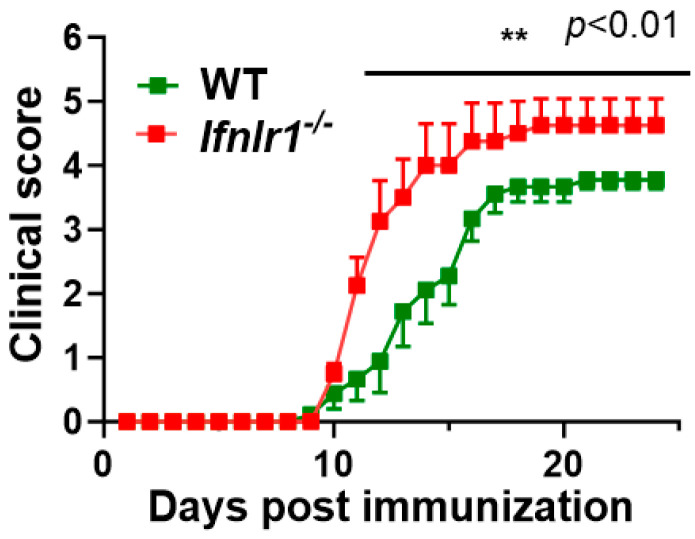
IFNLR1 knockout (*Ifnlr1^−/−^*) mice develop significantly more severe EAE compared to wild-type (WT) mice. EAE clinical score of *Ifnlr1^−/−^* (red, *n* = 10) and *WT* (green *n* = 10) mice immunized with MOG_35–55_ peptide in CFA. Mice were immunized with 150 µg MOG_35–55_ peptide in CFA and the clinical course of disease was determined. Mice with an EAE clinical score above 4 or a drop in weight of over 20% were humanely euthanized and removed from future analysis. Data represent mean ± SEM; ** *p* < 0.01.

As a first step to interrogate the role of IFN-λ in autoimmune neuroinflammation, we assessed the development and progression of EAE in WT and *Ifnlr1^−/−^* mice following immunization with MOG_35–55_ peptide (MOGp). EAE symptoms were monitored daily using a standard clinical score as described in the Methods section. The *Ifnlr1^−/−^* mice developed significantly more severe EAE than WT littermates (Figure 1).

**Figure 2:**
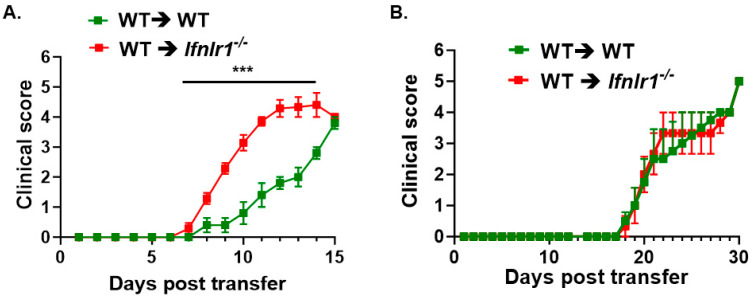
IFN-λ signaling plays an important role in restraining EAE induced by encephalitogenic Th1 cells but not for Th17 cells. For passive induction of EAE, MOG_35–55_ reactive Th17 or Th1 cells were generated from immunized donor C57Bl/6 mice. Ten days following immunization, cells from peripheral lymph nodes and spleen were restimulated with 10 μg/mL MOG_35–55_ under either Th17 or Th1 polarizing conditions for 3 days. These cells were used as “donor cells”. WT (*n* = 10) and *Ifnlr1*^−/−^ (*n* = 10) mice were injected with 2.5 × 10^6^ cells (Th17 (**A**) or Th1 (**B**)) resuspended in 500 μL of PBS via intravenous tail injections. Recipient mice were also intraperitoneally administered 200 ng of the pertussis toxin, the same as for active EAE, and they were scored daily to assess clinical symptoms of EAE for 30 days. Data represent mean ± SEM; *** *p* < 0.0001.

Previously, we demonstrated that type 1 IFN receptor (IFNAR) signaling was necessary to restrain EAE induced by encephalitogenic Th1 cells but not Th17 cells [16]. We, therefore, interrogated whether IFN-λ signaling differentially modulates disease induced by encephalitogenic Th1 and Th17 cells. *Ifnlr1^−/−^* recipient mice of encephalitogenic Th1 cells developed significantly more severe EAE compared to WT recipient mice (*p* < 0.001) (Figure 2A). The day of onset of EAE as well as peak of disease was similar for WT and *Ifnlr1^−/−^* recipients; *Ifnlr1^−/−^* recipients reached peak of disease by day 12, about three days earlier than WT recipients. In contrast to Th1 cells, encephalitogenic Th17 cells induced EAE of equal severity in WT and *Ifnlr1^−/−^* mice (Figure 2B).

### 3.2. IFN-λ Signaling in Macrophages Restrains the Expansion of Encephalitogenic Th17 Cells but Not Th1 Cells under Restimulation Conditions In Vitro

**Figure 3:**
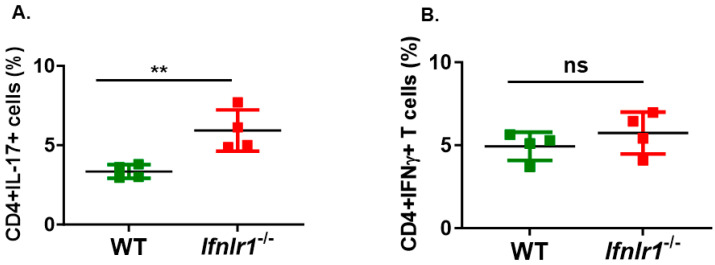
IFN-λ signaling in macrophages restrains the expansion of encephalitogenic Th17 cells but not Th1 cells. Encephalitogenic Th17 cell expansion by *Ifnlr1^−/−^* macrophages. The proportion of (**A**) IL-17+ or (**B**) IFN-γ+ CD4 T cells in cultures following 3-day restimulation with MOGp in the presence of peritoneal macrophages from WT or *Ifnr1^−/−^* mice. Each dot represents macrophages from an individual mouse. CD4 T cells were obtained from MOGp-immunized WT mice. Data represent mean ± SEM; ** *p* < 0.01; ns = not significant.

Considering that Th1-driven EAE but not Th17-driven EAE was aggravated in the absence of IFN-λ signaling, we tested the possibility that this represented differences in the function of IFN-λ signaling in antigen-presenting cells (APC) to regulate encephalitogenic Th1 vs. Th17 expansion. To address this possibility, we isolated CD4 T cells from spleens and lymph nodes of MOGp-immunized WT mice and restimulated them with MOGp using peritoneal macrophages from WT or *Ifnlr1^−/−^* mice as APC under Th17 or Th1 polarizing conditions. Under Th17 polarizing conditions, cultures containing *Ifnlr^−/−^* macrophages displayed a greater proportion of Th17 cells compared to those with WT macrophages (Figure 3A). In contrast, under Th1 polarizing conditions, WT and *Ifnlr1^−/−^* macrophages cultures contained similar proportion of Th1 cells (Figure 3B). From this data, it can be inferred that, in vitro, IFN-λ signaling in antigen presenting cells restrains the expansion of Th17 cells but not Th1 cells.

### 3.3. Neutrophil Populations (CD62L^lo^) in Spinal Cords Represent the Effector Population in EAE

**Figure 4:**
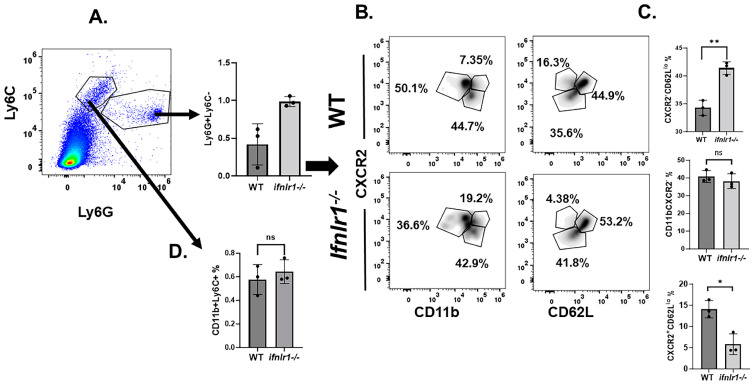
Neutrophil subpopulations in spinal cords of WT and *Ifnlr1* knockout mice with EAE. Single-cell suspension of perfused spinal cord from WT or *Ifnlr1^−/−^* mice with EAE were stained for expression of Ly6C, Ly6G, CD11b, CD62L, and CXCR2. Neutrophils were gated as Ly6G+Ly6C^lo^ (**A**) and then examined for expression of CXCR2 and CD11b or CXCR2 and CD62L (**B**). The proportion of Ly6G + Ly6C-, CD11b + CXCR2-, CXCR2-CD62L^lo^, and CXCR2 + CD62^lo^ in WT and *Ifnlr1^−/−^* mice (**C**). Macrophages were gated as CD11b^+^LyC^+^LyG^+^ cells (**D**). Total number of mice *n* = 3 of each genotype. * *p* < 0.05; ** *p* < 0.01; ns = not significant.

Neutrophils express IFNLR and it is known that its engagement by IFN-λ inhibits their migration and production of ROS [7,10]. Therefore, we postulated that the less severe EAE observed in *Ifnlr1* KO mice reflects the effect of IFN-λ on neutrophils. Consistent with this possibility, early in EAE disease (day 10 after immunization), the proportion of Ly6C^−^Ly6G^+^ cells representing neutrophils was significantly elevated in *Ifnlr1^−/−^ mice* compared to WT mice (Figure 4A). The majority of neutrophils in *Ifnlr1^−/−^* mice displayed CXCR2^−^CD62L^lo^ phenotype indicating effector neutrophil population (Figure 4B) [18]. The frequency of CXCR2^+^CD62L^hi^ was similar in WT and *Ifnlr1^−/−^* mice. Though present in smaller numbers, the frequency of CXCR2 + CD62L- neutrophils was significantly lower in *Ifnlr1^−/−^* mice with EAE (Figure 4B).

A recent study showed that the loss of IFN-λ signaling increased the frequency of inflammatory macrophages in CNS of EAE mice [14]. As depicted in Figure 4C, no difference in the frequency of macrophages was observed in early EAE in the presented disease model (CD11b^+^LyC^+^LyG^−^) (Figure 4C).

## 4. Discussion

In this report, we demonstrate that IFN-λ signaling is necessary to restrain autoimmune neuroinflammation severity in the EAE model of MS. We further showed that a transfer of encephalitogenic Th1 cells, but not Th17 cells, induced EAE with higher severity in naïve *Ifnlr1^−/−^* compared to WT mice.

In contrast to our findings, a recent study demonstrated that the *Ifnlr1^−/−^* mice developed less severe EAE than the WT mice [14]. The investigators conclude that IFN-λ signaling in myeloid cells, specifically in CD11c+ cells, was important in restraining EAE severity. The *Ifnlr1^−/−^* mice in this study are the same as those used by Manivasagam et al. [14]. Although we are unable to provide a definitive explanation for the underlying reasons for the disparate results between our two studies, a plausible explanation includes the differences in immunization approaches. Our immunization approach in C57Bl/6 leads to the development of chronic EAE with no recovery in disease severity (Figure 1). In the study by Manivasagam et al. [14], the *Ifnlr1^−/−^* mice developed chronic EAE; however, there was some degree of recovery. Observed differences in outcomes may reflect differences in experimental approaches including sites of immunization (upper back used here vs. flanks), immunization doses, and others, as previously reported for EAE outcome in *Ifnlr1^−/−^* mice [19,20,21,22].

Both adaptive and innate cells of the immune system contribute to the pathology of MS and EAE. Most studies in this field focused on the role of CD4+ T lymphocytes, which form part of the adaptive immune system as both mediators and regulators in disease pathogenesis [23]. Recent studies have suggested that the innate immune system also plays a vital role in the initiation and progression of MS by influencing the effector function of T and B cells [24]. The cells of the innate immune system can prevent autoimmunity by differentiation of regulatory T cells and by secretion of neurotrophic growth factors. The innate immune system can also play a pathogenic role by promoting the differentiation of Th1 and Th17 cells that drive acute inflammatory events associated with relapses in MS [24]. The effector cells express cytokines and activation markers that further activate innate immune cells [25]. Furthermore, innate immune cells have been implicated in the progressive phase of MS, as reflected by their activated phenotype in the periphery, which might be responsible for neurodegenerative changes in secondary progressive MS.

Several studies in EAE models indicate that neutrophils play an essential role in inflammation by producing cytokines and damaging the blood–brain barrier (BBB). Neutrophils can influence the manifestation of EAE by facilitating parenchymal brain inflammation [26]. Neutrophils are necessary and sufficient to induce demyelination in mouse models of MS [27,28]. Neutrophil expansion in the periphery and CNS is an early event in EAE and their depletion leads to delayed onset and attenuated disease [29,30,31,32]. Demyelination and axonal damage are directly orchestrated by autoreactive T cells, B cells, neutrophils, macrophages, and microglia, and indirectly through neutrophil-mediated release of pro-inflammatory factors including ROS, nitric oxide, matrix metalloproteinases (MMPs), and IL-1β [33,34,35]. While expansion of neutrophils early in EAE is linked to the expression of granulocyte-colony stimulating factor (G-CSF) [36], their recruitment into the CNS (brain and spinal cord) is dependent on Th1 and Th17 chemokines, CXCL1, CXCL2, and CXCL6, that activate the CXCR2 receptor [32,36,37]. Neutrophils are localized in active MS lesions at the sites of BBB leakage and actively contribute to BBB permeability [30,32,38,39]. Neutrophils mediate BBB breakdown via contact-dependent mechanisms and through the secretion of enzymes, myeloperoxidase (MPO), matrix metalloproteinases (MMPs), and ROS [40]. *Mmp2*^−/−^ and *Mmp9*^−/−^ double knockout mice are resistant to EAE development [41]. In MS patients, an increase in MMP-9 levels in CSF and blood correlates with disease activity [42,43]. MPO increases BBB permeability and neutrophil migration into the CNS [44] and induces the production of highly reactive ROS resulting in neuronal damage [45]. A neutrophil subset, polymorphonuclear myeloid-derived suppressor cells (PMN-MDSCs), plays a critical role in immune regulation [46,47] and was shown to ameliorate EAE through suppression of B cells, Th1, or Th17 cells via programmed death ligand 1 (PD-L1)/PD-1 interaction [48,49,50,51,52,53].

We found that *Ifnlr1^−/−^* mice exhibited an increased proportion of early-infiltrating neutrophils but not macrophages compared to WT mice. These neutrophils were predominantly CXCR2^−^CD62^lo^, a population of activated neutrophils [18]. In EAE, neutrophils comprise a significant percentage of CNS-infiltrating cells prior to disease onset and relapse [26]. Disease was ameliorated when neutrophils were depleted prior to, but not after, the onset of disease or relapse, suggesting their essential function during the early steps of autoimmune neuroinflammation [26,36]. IFN-λ signaling exerts a regulatory function on neutrophils in MS, SLE, and RA [3,7,11,12,13]. The signal from IFN-λ regulates neutrophil activity via the inhibition of migration, ROS, NETosis, and expression of IL-1*β* and transcription factor IRF1 [3,4,5,6,7,8,9,10]. We also noted that IFNLR1-deficient macrophages are more efficient in promoting expansion of Th17 effector cells. Il-17A functions as a chemokine to promote recruitment of neutrophils and this could represent a mechanism for the greater numbers of neutrophils early during EAE in *Ifnr1^−/−^* mice [54]. Currently, the precise mechanism underlying the increased EAE severity in *Ifnlr1^−/−^* mice is unclear. Based on the findings presented here, we predict that a key property of IFN-λ is its ability to inhibit neutrophil migration into the CNS. This possibility is supported by a preliminary experiment using positron emission tomography (PET) and translocator protein (TSPO) as a tracer, which revealed an increase in early neutrophil migration into the CNS (spinal cord and brain).

In our previous study, we demonstrated that type 1 IFN (IFNAR) signaling was crucial in restraining EAE induced by encephalitogenic Th1 cells but not Th17 cells [16]. This current study further supports these findings, as we observed that IFNLR1 signaling is also necessary to restrain EAE induced by encephalitogenic Th1 cells but not Th17 cells (Figure 2). This was somewhat unexpected since, in vitro, *Ifnlr1^−/−^* macrophages promoted the expansion of in vivo-primed MOGp-reactive Th17 cells but not Th1 cells. The difference between in vivo and in vitro data may reflect the combined effect of other factors that are active in vivo but are not fully replicated in vitro. It is feasible that IFNLR signaling restrains Th17 plasticity to Th1 cells or other aspects of Th17 pathogenicity [17,55,56].

IFN-β is an important therapeutic option for MS and remains an important comparator for newer treatments for this disease. IFN-λ has a distinct advantage over type I IFNs (IFNα/β) as its receptor has restricted expression on cells and tissues. Additional research is required to fully understand the context-dependent effects of IFN-λs for optimization of treatment in MS and other autoimmune diseases.

Overall, our studies highlight the crucial role of type III interferon, IFN-λ signaling in restraining autoimmune neuroinflammation, potentially through the inhibition of neutrophil migration and/or activation. Importantly, our findings also suggest that IFN-λ exerts pleiotropic effects in EAE and MS which are context-dependent and may be anti-inflammatory or inflammatory, as previously demonstrated for IFN-γ [19].

## Data Availability

The data presented in this study are available on request from the corresponding author.
